# Sequential MALDI-MSI-Based Multiomics Reveals Spatial Lipid, Glycan, and Tryptic Peptide Signatures in Breast Tumor Histopathology

**DOI:** 10.1021/acs.analchem.6c01243

**Published:** 2026-05-06

**Authors:** Seyed M. J. Seyed Golestan, Nicole Monza, Farnaz Fatahian, Lisa Pagani, Mohammad Ali AS‘habi, Hossein Behboudi, Andrew Smith, Alireza Ghassempour, Vanna Denti

**Affiliations:** † Medicinal Plants and Drug Research Institute, 556492 Shahid Beheshti University, Tehran 1983969411, Iran; ‡ 9305 University of Milano Bicocca, Department of Medicine and Surgery, Proteomics and Metabolomics Unit, Vedano al Lambro, 20854, Italy

## Abstract

The high molecular heterogeneity of breast cancer (BC) poses a significant challenge for its classification and biological characterization. Despite numerous efforts, conventional immunohistochemical techniques and traditional mass spectrometry (MS) have failed to provide an exhaustive characterization of tumor subtypes. This limitation is likely due to the loss of spatial information, which significantly impacts the interpretation of the results. In this study, we present a matrix-assisted laser desorption/ionization-mass spectrometry imaging (MALDI-MSI) approach that spatially integrates three multiomics layers, including lipids, N-glycans, and tryptic peptides, on the same tissue microarray (TMA) section with BC and normal tissue cores. The analysis of individual layers and their integration demonstrates the potential of multiomics MALDI-MSI in discriminating between healthy and tumor tissues and in capturing molecular differences associated with different subtypes of BC. Specifically, the approach adopted highlighted the significant contribution of lipids and glycans to characterizing breast tumor subtypes. The proteomic layer provides complementary information on the proliferative state and biological heterogeneity of the tumors, clearly distinguishing between the healthy and neoplastic conditions. Overall, this proof-of-concept study demonstrates the potential of spatial multiomics MALDI-MSI as a tool for a more in-depth characterization of BC subtypes, laying the groundwork for future applications on larger sample cohorts.

## Graphical abstract

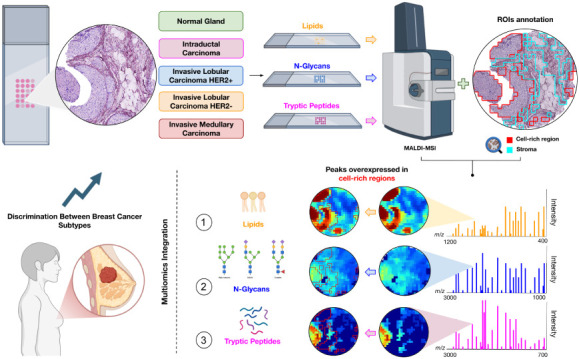

## Introduction

Breast cancer (BC) is a biologically heterogeneous disease that affects mostly women worldwide. Its clinical behavior and therapeutic response are governed by complex, spatially organized molecular programs that extend beyond conventional histopathological stratification.^1,2^ Although conventional diagnostic frameworks primarily rely on histopathological evaluation and immunohistochemical assessment of hormone receptor and human epidermal growth factor receptor 2 (HER2) expression, these approaches often fall short in capturing the extensive biological and morphological heterogeneity that characterizes BC.^2^ This heterogeneity is particularly evident across distinct histopathological architectures, such as intraductal (IC),^3,4^ invasive lobular (ILC),^5^ and invasive medullary carcinomas (IMC),^6,7^ which exhibit diverse growth patterns, stromal interactions, and degrees of invasiveness, ultimately influencing prognosis and therapeutic response.

Despite their routine clinical use, traditional classifications lack the molecular depth needed to resolve the biochemical complexity underlying the histotypes. In particular, spatial variations in metabolic pathways, glycosylation patterns, and proteomic composition remain poorly characterized, limiting our understanding of how these features contribute to tumor progression and treatment resistance. Advances in high-throughput technologies have revealed extensive alterations across multiple molecular layers, highlighting the complexity of biochemical reprogramming in BC. In this context, mass spectrometry (MS)-based approaches have been increasingly applied to characterize tumor heterogeneity through molecular-level profiling of lipids, N-glycans, and proteins in BC.^8–13^ In this context, lipidomic analyses have revealed dysregulated lipid metabolism across BC subtypes, with phosphatidylcholines (PC) and ceramides (Cer) linked to BC aggressiveness,^14–17^ while sphingomyelins (SM) have been associated with aggressive tumor progression.^18^ Similarly, N-glycomic alterations can facilitate tumor progression by modulating immune evasion and metastatic potential,^19^ whereas proteomics analysis further refines molecular subtyping by identifying differentially expressed proteins that influence prognosis and therapeutic response.^20–22^

Despite these advances, conventional MS-based omics workflows largely rely on bulk measurements, thereby obscuring spatial heterogeneity and molecular interactions within the tumor microenvironment (TME). Indeed, the presence of stroma, blood vessels, and immune system cells in the TME contributes to BC heterogeneity and complexity, representing a valuable source of information for treatment options.^23–25^ Matrix-assisted laser desorption/ionization mass spectrometry imaging (MALDI–MSI) addresses this limitation by enabling label-free, spatially resolved mapping of diverse biomolecular classes directly within formalin-fixed paraffin-embedded (FFPE) tissue sections while preserving histological context.^26–28^ Building on this potential, recent work by Denti et al. demonstrated the feasibility of performing sequential MALDI-MSI of lipids, N-glycans, and tryptic peptides on a single slide, providing a spatially resolved multiomics view of cancer subtypes.^28^ Histopathology-driven characterization distinguishing normal gland, IC, ILC, and IMC offers a biologically grounded framework that better reflects tissue architecture and clinical diagnosis.

In this study, we applied sequential MALDI-MSI spatial multiomics to integrate lipidomic, N-glycomic, and tryptic peptide layers across normal glands, ICs, ILCs, and IMCs, as well as further differentiating HER2-positive and HER2-negative lobular subtypes. This proof-of-concept study enables molecular characterization directly within defined tissue architectures and reveals coordinated metabolic and structural signatures that bridge morphology with molecular organization, advancing precision pathology in BC.

## Experimental Procedures

### Chemicals and Reagents

High-performance liquid chromatography (HPLC)-grade toluene, HPLC-grade methanol (MeOH), HPLC-grade acetonitrile (ACN), HPLC-grade ethanol (EtOH), and HPLC-grade water (H_2_O) were obtained from Honeywell SC, Seelze, Germany. Liquid chromatography–mass spectrometry (LC–MS) grade formic acid (FA), ACN, and H_2_O were acquired from LiChrosolv, Merck KGaA, Darmstadt, Germany. Trifluoroacetic acid (TFA), citric acid, phosphorus red (PR), guanidinium chloride (GUA), diammonium hydrogen citrate (DAHC), ammonium bicarbonate (NH_4_HCO_3_), DL-dithiothreitol (DTT), iodoacetamide (IAA), PNGase F from *Elizabethkingia meningoseptica*, and trypsin derived from porcine pancreas were purchased from Sigma-Aldrich, Buchs, Switzerland. RapiGest SF Surfactant was purchased from Waters Corporation, Milford, MA, USA. The α-Cyano-4-hydroxycinnamic acid (CHCA) matrix and PepMix I were sourced from Bruker Daltonics, Bremen, Germany, and the 6-Aza-2-Thiothymine (ATT) matrix was provided by ChemCruz, Santa Cruz, CA, USA.

### Specimen Selection

The FFPE samples used in this work to create a BC peptide library included blocks of five anonymized *ex vivo* human breast tumor tissue sections obtained from Imam Khomeini Hospital (Tehran, Iran), following the ethics committee standards of Shahid Beheshti University of Medical Sciences (IR.SBMU.PHARMACY.REC.1403.032). These samples belonged to five different patients, all diagnosed with grade 3 breast cancer. The average size of the whole tumors was approximately 2.5 × 1.3 × 0.7 cm. Based on pathology reports, the tumor subtypes included IC, ILC, and IMC.

Spatial multiomic analyses were performed on a 5 μm-thick TMA purchased from Tissuearray.com (https://www.tissuearray.com/, code: BR1503h) of different types of human BC and normal breast tissue (Derwood, MD, 20855, USA) placed on a conventional glass slide for immunohistochemistry (IHC).

### Sample Preparation

#### Sample Preparation for MALDI-MSI of Lipids

For spatial lipidomics analysis, the ITO slide was initially heated at 65 °C for 1 h to premelt the paraffin, and subsequently, deparaffinisation was carried out with three consecutive toluene washes of 5 min each. Then, ATT^29^ (10 mg/mL, 70:30 MeOH:H_2_O, 2 mM GUA, 20 mM DAHC) was applied to the slide using an HTX TM-Sprayer (HTX Technologies, LLC) under the conditions outlined in Table S1. Before performing MALDI-MSI analysis, Phosphorus Red (PR) was spotted onto the slides as a calibration standard.

#### Sample Preparation for MALDI-MSI of N-Glycans

After performing MALDI-MSI on lipids, the ATT matrix was removed from the slide with absolute EtOH, followed by a rehydration process involving sequential washes in 100% EtOH (1 × 3 min), 70% EtOH (1 × 3 min), and HPLC H_2_O (2 × 2 min). A citric acid antigen retrieval procedure was carried out by immersing the slides in a citrate buffer (pH 5.9, 10 mM) at 97 °C for 45 min. This was followed by a 20-min wash in water to prepare the slides for enzymatic treatment. The PNGase F enzyme (2 U/mL) was applied using an automated iMatrixSpray system (Tardo GmbH, Subingen, Switzerland), adhering to the parameters outlined in Table S2. The slides were then incubated overnight (for approximately 18 h) in a humidity chamber at 42 °C for digestion. Finally, a solution of CHCA (5 mg/mL, 70:30 ACN:H_2_O, 1% TFA) was deposited onto the slides using the HTX TM-Sprayer, following the optimized conditions detailed in Table S1.

#### Sample Preparation for MALDI-MSI of Tryptic Peptides

After conducting MALDI-MSI of N-glycans, the CHCA was removed from the slide, and rehydration was carried out as outlined in the previous section. Trypsin was applied at a concentration of 20 ng/μL using the iMatrixSpray automated spraying system, adhering to the parameters listed in Table S2. The samples were then placed in a humidity chamber at 40 °C overnight. Finally, a solution of CHCA (10 mg/mL, 70:30 ACN:H_2_O, 1% TFA) was sprayed onto the samples using the HTX TM-Sprayer, following the optimized conditions specified in Table S1.

### MALDI-MSI Parameters

All MALDI-MSI analyses of the TMA were performed using a timsTOF fleX mass spectrometer (Bruker Daltonics, Bremen, Germany), equipped with a Smartbeam 3D laser operating at a frequency of 10 kHz. All acquisitions were performed in reflectron positive ion mode. For spatial lipidomics, mass spectra were acquired in the mass range *m*/*z* 500–1200, while N-glycan and tryptic peptide analyses were performed in the mass range of *m*/*z* 1000–3000 and 700–3000, respectively. External calibration for lipids was achieved using PR clusters (*m*/*z* range 0–2000), while N-glycan and tryptic peptide calibration was performed using PepMix I (Bruker Daltonics), applied directly to the TMA slide before the MALDI-MSI analysis.

The acquisition parameters were configured using timsControl software (v.2.0.51.0_9669_1571, Bruker Daltonics), with a beam scan setting of 46 μm and a raster width of 50 × 50 (*x*, *y*).

### Hematoxylin and Eosin Staining and QuPath Pixel Classifier

After spatial proteomics analysis, the matrix was removed by washing the slide with increasing concentrations of EtOH (70% and 100%) and then stained using hematoxylin and eosin (H&E). The slides were then digitized using a MIDI II digital scanner (3DHISTECH, Budapest, Hungary), enabling the integration of the morphological and proteomic data by overlapping the images. Subsequently, the H&E-scanned image was imported into QuPath open-source software (https://qupath.github.io/), where a trained pixel classifier was applied to pinpoint specific cell-rich regions of interest (ROIs) of breast tissue and remove stroma and hole regions, as previously described.^30^

### nLC-ESI-MS/MS Sample Preparation

Five FFPE BC tissue sections, each representing a distinct molecular subtype, were pooled for protein identification and annotation using nanoelectrospray ionization liquid chromatography tandem mass spectrometry (nLC-ESI-MS/MS). The pooled sample underwent deparaffinization, antigen retrieval, and enzymatic digestion following the protocol described by Principi L. et al.,^31^ with small modifications. Briefly, the sample was incubated for 1 h at 65 °C, followed by consecutive washes (5 min while sonicating) and centrifugation (14,000 rpm, 5 min) cycles with toluene (3 times), 100% EtOH (2 times), 90% EtOH, 70% EtOH, and water. Heat-induced antigen retrieval using citric acid (10 mM, pH 6) at 97 °C for 45 min was performed, followed by centrifugation (14,000 rpm, 5 min). The sample was washed with water and then centrifuged (14,000 rpm, 5 min). Subsequently, it was resuspended in 50 mM buffer solution containing ammonium bicarbonate and 0.1% RapiGest SF surfactant and incubated for 15 min at 60 °C while shaking. Reduction and alkylation of disulfide bonds were performed by adding DTT 10 mM (incubation at 56 °C for 45 min while shaking) and IAA 15 mM (incubation in the dark at room temperature for 30 min). Proteins were digested by adding 5 μg of trypsin and incubated overnight at 37 °C. Enzymatic activity was quenched by adding TFA to a final concentration of 0.5%, lowering the pH to below 2. RapiGest SF surfactant removal was operated by incubating the samples for 30 min at 37 °C, followed by centrifugation at 13,000 rpm for 10 min. The supernatant containing peptides was collected, dried using a vacuum centrifugal evaporator (Hetovac, Savant), and reconstituted in 50 μL of 0.1% formic acid. Peptide concentrations were determined using a NanoDrop spectrophotometer (Thermo Scientific, Sunnyvale, CA).

### nLC-ESI-MS/MS Analysis

An amount of 500 ng of tryptic peptides was analyzed in duplicate using a Dionex UltiMate 3000 RSLC nano system (Thermo Scientific, Sunnyvale, CA, USA), coupled online with a timsTOF fleX mass spectrometer (Bruker Daltonics, Bremen, Germany) equipped with a CaptiveSpray source. The samples were loaded into a μ-precolumn (Thermo Scientific, Acclaim PepMap 100, 100 μm × 2 cm, nanoViper, C18, 3 μm) for a further desalting and concentration step; then, the peptides were separated in an analytical 50 cm nanocolumn (Thermo Scientific, Acclaim PepMap RSLC, 75 μm × 50 cm, nanoViper, C18, 2 μm) using a 90-min multistep gradient ranging from 4% to 98% of mobile phase B (H_2_O:ACN:FA, 20:80:0.08) at a flow rate of 300 nL/min and a temperature of 40 °C. The mass spectrometer was operated in DIA (Data-Independent Acquisition)-PASEF (Parallel Accumulation-Serial Fragmentation) mode, following parameters adapted from recent publications.^32,33^ Ions were scanned in positive mode over an *m*/*z* range of 100–1700 and a mobility range of 0.60–1.60 V·s/cm^2^. Dry gas flow was 3.0 l/min at 180 °C, and capillary voltage was 1650 V. For tandem mass PASEF analysis, the cluster of monocharged ions was excluded to reduce the complexity of MS2 spectra using the following parameters: mass range 300–1201 Da and 0.60–1.43 1/K_0_. The estimated cycle time for each PASEF analysis was 1.8 s, with a total of 16 cycles using DIA windows of 25 Da.

The mass spectrometer was calibrated for mass accuracy using a mix of ten standards with known mass (MMI-L Low Concentration Tuning Mix, Agilent Technologies, Santa Clara, CA, USA). With the nanosource, mass and ion mobility calibration was performed using three specific lock masses (622.0290 *m*/*z*, 922.0098 *m*/*z*, and 1221.9906 *m*/*z*) applied to a filter.

### nLC-ESI-MS/MS Data Analysis

Raw data were processed using the PEAKS Studio 12.5 platform (Bioinformatics Solutions Inc., Waterloo, ON, USA), which employed a library-free processing method based on the human SwissProt database (downloaded in September 2024). The parameters for the analysis included trypsin as the enzyme; carbamidomethylation (C) as a fixed modification; FFPE + 12 and +30, acetylation (protein N-term), and oxidation (M) as variable modifications. The criteria for identification included a peptide-level FDR of 1% and a −10 log P score of ≥ 13 at the protein level, with only proteins containing at least one unique peptide considered as identified. The identified proteins were used to create a protein data set to match with MALDI-MSI-derived *m*/*z* features.

### MALDI-MSI Data Processing and Putative Annotations

The data obtained from the MALDI-MSI analysis were consolidated into three different files using SCiLS Lab 2026b software (http://scils.de/; Bremen, Germany). Baseline subtraction was performed using the convolution algorithm, followed by root-mean-square (RMS) normalization. The coregistration of the MSI signals and the H&E-stained image is performed with the registration tool on SCiLS Lab software, where corresponding anatomical features were manually selected on both images.

A comprehensive list of *m*/*z* features for the single three layers was then generated using the “Feature Finding” tool, applying the T-ReX^2^ (QTOF) algorithm, weak spatial noise filtering, and a coverage of 35%. The obtained feature list was then imported into mMass software (version 5.5.0, http://www.mmass.org), where peak picking was performed.

For the spatial lipidomics data set, the *m*/*z* feature list created was tentatively annotated using MetaboScape 2025 software (https://www.bruker.com; Bremen, Germany). The putative lipid species annotation section with the default lipid database, comprehensive of glycerolipids, glycerophospholipids, sphingolipids, and sterol lipids, was used for the annotation considering ion notations [M]+, [M+HH]+, [M + Na]+, and [M+K]+ and a mass error of ± 10 ppm (ppm). A total of 34 features were tentatively annotated and reported in Table S3.

The *m*/*z* feature list of the glycomic data set was imported into the Metaspace open-source software^34^ (https://metaspace2020.org/) using the NGlycDB-v1 database and a mass error of ± 10 ppm. A total of 45 features were putatively annotated and reported in Table S4.

For the proteomics data set, the nLC-ESI-MS/MS-created library was matched with MALDI-MSI-derived *m*/*z* features, considering a mass error of ± 10 ppm. More than one putative annotation was obtained for most of the *m*/*z* features. The features list was narrowed down based on the lower value of error in ppm, resulting in a total of 306 unique putative *m*/*z* signals that are reported in Table S5.

Any *m*/*z* signals without an assigned identity were excluded from the lists and statistical analysis. Ultimately, a refined, integrated feature list combining the annotated *m*/*z* features from the three omics layers was used for the multiomics analysis.

### Statistical Analysis

The statistical analyses were performed using MetaboAnalyst 6.0 (http://MetaboAnalyst.ca/), an open-source platform for the statistical evaluation of omics data sets. No data normalization was performed on the data sets. To identify the most significantly altered lipids, N-glycans, and tryptic peptides, a one-way analysis of variance (ANOVA) was conducted considering a *p-value* < 0.001, with Fisher’s LSD post hoc analysis and a raw *p-value* cutoff of 0.05, adjusted for multiple comparisons using the Benjamini–Hochberg method. The significantly varied lipids, N-glycans, and tryptic peptides were then compiled into a table to assess their abundances in BC subtypes.

Furthermore, principal component analysis (PCA) and hierarchical clustering heatmaps of the single layers and the multiomics integration were generated to better understand the data. For the lipidomic data set, the ILC HER2+ and HER2- comparison was integrated with an unpaired volcano plot analysis (*p-value* < 0.05) to better select significant features based on either biological or statistical significance. For the proteomic data set, a hierarchical clustering dendrogram tree was included, considering the Pearson distance measure and the Ward clustering algorithm. Moreover, a correlation heatmap using Pearson’s r as a distance measure was performed in the multiomics integration to visualize the correlation among the different classes of features.

## Results and Discussion

### Clinicopathologic Characteristics of the Study Cohort

The clinicopathological characteristics of the BC TMA cores analyzed in this study are summarized in Table S6. The cohort included normal breast tissue (n = 4) and three histologically distinct tumor entities representing progressive stages of disease evolution: IC, ILC, and IMC, with four cores per subtype. Immunohistochemical profiling included estrogen receptor (ER), progesterone receptor (PR), HER2, and the Ki67 proliferation index. Tumor staging was assigned according to the tumor, node, metastasis (TNM) classification system. To minimize interindividual variability and ensure consistency across molecular layers, all samples were selected according to predefined clinical criteria, including a restricted age range (40–50 years), tumor stage (0–IIIB), and receptor expression profiles.

### Lipidomic Profiling Reveals Subtype-Specific and HER2-Dependent Molecular Heterogeneity in Breast Cancer

As an initial exploratory step, we investigated lipidomic alterations associated with BC progression by using MALDI-MSI. Lipids are central regulators of membrane organization, signaling, and cellular stress adaptation; therefore, their spatial distribution may provide a direct biochemical readout of BC tissue architecture. The analysis of healthy tissue and tumor conditions shows partially overlapping lipidomic profiles, although the more aggressive invasive form appears to differentiate more (Figure S1A). To better investigate the molecular alterations underlying the histopathological and phenotypic diversity of BCs, subsequent analyses focused solely on tumor conditions, with particular attention to HER2-driven stratification within the ILC (ILC HER2+ and ILC HER2-). PCA demonstrates a clear separation between all four tumor lesions, suggesting a separation across the BC subtypes (Figure S2A). The first three principal components captured the vast majority of the variance, supporting discrimination among BC tissue phenotypes. Conversely, the hierarchical clustering heatmap of the top 20 features based on the ANOVA highlights a higher expression of most of the putative SM, long-chain PC, and glycosylated ceramide (GlcCer) and a lower expression of lysophosphatidylcholines (LysoPC) in the most aggressive and invasive forms of BCs (Figure 1A). On the basis of the ANOVA results, Figure 1B shows four of the most representative lipids among the 20 significant ones. These box plots, based on the Pixel Classifier annotations, support the subtype-specific lipid trends, showing higher SM levels in IMC compared to the less aggressive lesions. Conversely, a higher intensity is observed for LysoPC (18:00) and LysoPC (18:01) in in situ carcinoma (IC), followed by ILC-HER2+ and IMC, respectively (Figure 1B).

**Figure 1:**
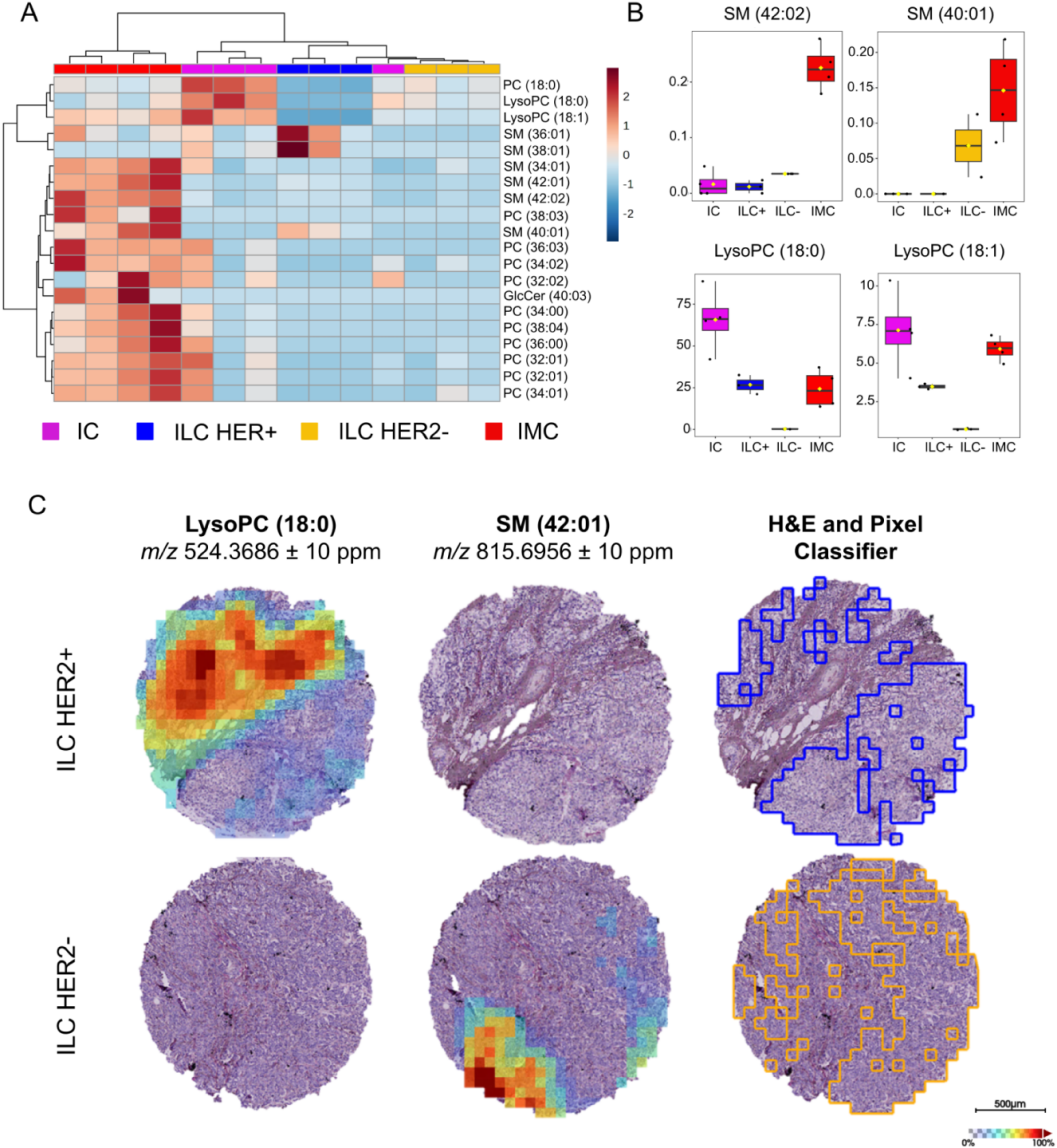
Lipidomic characterization of breast cancer subtypes and HER2-stratified ILC: A. Hierarchical clustering of the top ANOVA-selected lipids reveals subtype-specific lipid signatures. B. Pixel Classifier ROI-based boxplots of representative lipids show increased sphingomyelins in IMC and higher LysoPC levels in IC and ILC HER2+. C. MALDI-MSI spatial distribution of LysoPC (18:0) and SM (42:01) overlaid on H&E-stained sections and pixel classification highlights the association of lipid signatures with tumor and stromal regions. Weak denoising applied.

A detailed analysis of this subtype suggests a different pattern of lipid expression between ILC-HER2+ and ILC-HER2- tumors. The volcano plot shows a prevalence of sphingolipid-significant species among ILC-HER2-, whereas the phospholipid class appears to dominate ILC-HER2+ (Figure S2B). Moreover, the additional heatmap (Figure S2C) and the spatial distribution of two representative lipids among the two ILC classes (Figure 1C) are consistent with this observation. These lipid classes display a heterogeneous spatial distribution within tumor regions, highlighted in blue and orange, and localize between tumor and stromal areas (Figure 1C). LysoPC is generated from PC intracellularly by phospholipase A2, and they are known to colocalize in the plasma membrane.^35^ A recent study using DESI-MSI demonstrated a correlation between PC expression and its spatial localization in cancer and partially in the stroma of BC,^35^ which is consistent with our results.

The lipid expression pattern observed in Figure 1A is generally consistent with the distinctive biological behavior of invasive vs in situ BC subtypes. On the other hand, ILC-HER2+ shows lipid features partially overlapping with IC, whereas ILC-HER2– appears to share similarities with IMC for selected lipid classes (Figure 1A and 1B). Overall, choline-containing lipid and sphingomyelin downregulation appear to be evident in the ILC classes.

The different abundance intensities of SMs and LysoPCs within the cancer subtypes are in line with their biological roles. SM represents the most abundant lipid of the plasma membrane and has been demonstrated to induce cancer growth and progression by modulating cell proliferation and migration.^36^ Increased levels of SM are known to be associated with cancer immune evasion, which is typical of BC invasive behavior.^37^ Whereas a higher expression of LysoPCs is observed in ILC-HER2+ compared to the HER2- condition, their expression appears to be absent. This observation suggests that HER2 could contribute to lipidomic alterations within the ILC class.

Overall, our lipidomic study shows a potentially distinct molecular stratification of BC subtypes, including fine-grained HER2-based discrimination within the ILC class. Increased levels of putative SM and long-chain PC appear to be associated with the most aggressive and invasive cancer morphologies, including IMC and ILC HER2, according to the spatially resolved lipid profiles. Similar alterations in these lipid species have been previously observed in BC tissues relative to adjacent normal tissue in MS and MSI studies.^38–41^ Conversely, increased levels of phospholipids appear to characterize IC and ILC HER2+ lesions, suggesting possible lipid remodeling associated with early-stage disease and specific molecular contexts. Although categorized as a singular histopathological entity, ILC appears to exhibit significant lipidomic heterogeneity in this subset of lipids, with HER2 expression potentially acting as a regulator of lipid composition and spatial distribution. The reduced abundance of these putative lipid classes in ILC, which resembles that of a more conventional lipid profile, may reflect its unique cellular architecture.

These findings indicate that lipidomic signatures may provide an additional layer of molecular classification in BC, especially within ILC, where traditional histopathological markers may not fully capture tumor heterogeneity.

### Glycomic Profiling Reflects Tumor Maturation and Invasive Progression in Breast Cancer

To investigate the glycomic changes associated with histopathological progression in BC, sequential MALDI–MSI was used to map the composition and spatial distribution of N-glycans across the same FFPE TMA that was previously analyzed by lipidomics. Descriptive analysis of the glycomic data revealed a clear separation between the healthy tissue and the tumor subclasses (Figure S1B) that appeared to be less evident in the lipidomic layer. Focusing exclusively on the tumor tissues, the hierarchical clustering heatmap based on the top 25 significant features for ANOVA (*p-value ≤ 0.001*) distinguished the invasive tumor subtypes (ILC and IMC) from the in situ carcinoma (IC), with the exception of one IMC replicate clustering closer to IC (Figure S3A).

To further characterize the glycan alterations underlying this separation, the intensity distributions of three representative glycans, among those identified as statistically significant for the ANOVA, are shown in Figure 2. Specifically, the complex glycans that were putatively identified as Hex:4HexNAc:3dHex:1 (*m*/*z* 1460.4810, fucosylated) and Hex:5HexNAc:4dHex:1NeuAc:1 (*m*/*z* 2116.7087, fucosylated and sialylated) showed higher intensity in IC, as seen in the pixel classifier ROI-based boxplots. The two complex glycans appear to colocalize within common stromal regions, whereas the fucosylated one seems to partially extend into the tumor-rich area defined using the automatic annotation described in the Methods section.

**Figure 2:**
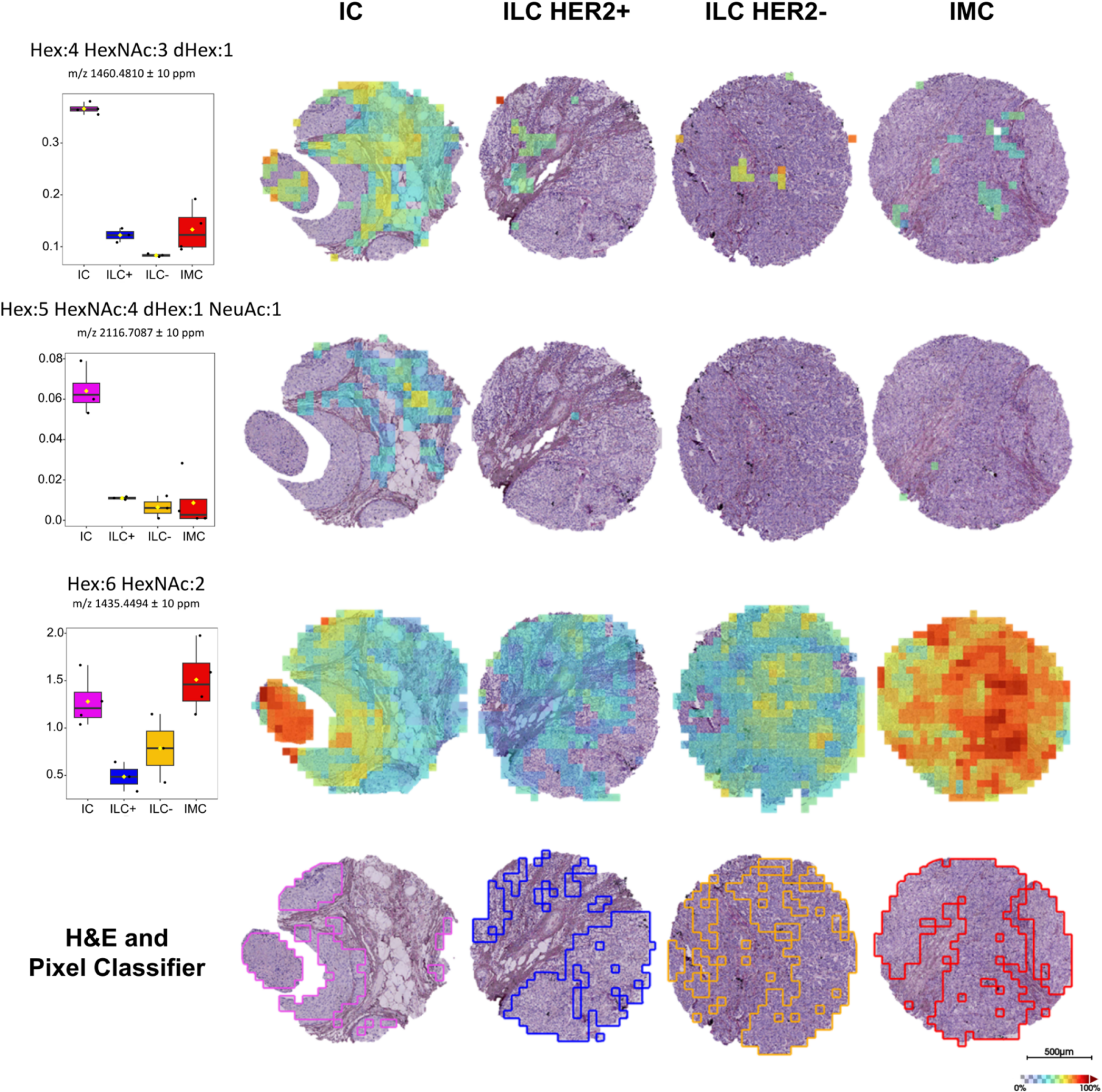
N-glycomic characterization of BC subtypes. The absolute intensity of three representative ANOVA-selected N-glycans is reported in the boxplot (on the left) with its spatial distribution across the four BC subtypes. The H&E and pixel-classifier-based annotation for each condition is reported at the bottom. Color scale: transparent (less intense) and red (more intense). Weak denoising applied.

In contrast, the high-mannose glycan putatively annotated as Hex:6HexNAc:2 (*m*/*z* 1435.4494) appears to be more abundant both in the tumor (defined by the pixel classifier) and stroma regions of IMCs and ICs. Contrary to what was observed in the lipidomic layer, the glycomic profile does not clearly separate ILC tumors based on HER2 status. Specifically, the receiver operating characteristic (ROC) analysis conducted solely on the two ILC subgroups identified a single complex glycan, putatively annotated as Hex:5HexNAc:4 (*m*/*z* 1679.5553), as potentially discriminating between ILC-HER2+ and HER2-, with an area under the curve (AUC) value of 0.8 and higher intensity observed in the HER2^–^ ILC subgroup, as shown in Figure S3.

Overall, complex glycan expression levels, including fucosylated and sialylated ones, appeared to be higher in the noninvasive condition, whereas signals putatively associated with high-mannose glycans were more represented in the most aggressive and invasive tumors (IMC). While these molecular assignments should be interpreted with caution, this observation is in line with previous reports across different cancer types, where high-mannose structures have been associated with immature glycoproteins arising from elevated protein synthesis, metabolic stress, and accelerated cellular proliferation.^42–45^ Accordingly, these glycans result to be present but less dominant in IC, while remaining generally low in ILC, which may match its discohesive growth pattern and metabolically less active phenotype; however, further validation is required to confirm these interpretations.

In line with their spatial distribution, the stromal enrichment of fucosylated glycans has been previously reported in MALDI-MSI studies and may be associated with extracellular matrix components.^46^ Similarly, high-mannose glycans are known to be markedly elevated in tumor-cell-rich regions, since they have been suggested to reflect increased protein turnover and endoplasmic reticulum stress associated with aggressive and invasive growth.^44^ However, a recent study demonstrates their presence in the tumor interstitial fluids (TIF) of the BC stroma, which may help explain the Hex:6HexNAc:2 localization in that area.^47^

### Proteomic Profiling Distinguishes Healthy and Tumor Breast Tissues and Reveals Ki67–Associated Heterogeneity in ILC

Following the glycomics analysis, the proteomic layer was examined using the MALDI-MSI approach. This allowed for a detailed characterization of protein expressions and interactions within the biological samples. Moreover, the nLC-MS/MS analysis of human BC samples allowed the identification of 22549 peptides and 4788 proteins. This list of peptides was matched with the *m*/*z* signals that emerged from MALDI-MSI investigation to allow putative annotations.

From the initial descriptive analysis of the MALDI-MSI data, including both healthy and tumor conditions, a clear separation between the two classes emerges, in agreement with the glycomic layer. In this case as well, higher protein expression is evident in the healthy condition compared to the tumor ones (Figure S1C), suggesting a global downregulation of most of the proteins identified during tumor development and increasing aggressiveness.

Considering only tumor conditions, similarities and differences between the various neoplastic components become evident. As before, the ILC condition was further subdivided on the basis of HER2 stratification. The hierarchical clustering identifies IC as a distinct group, while ILC samples distribute between IC and IMC, probably according to their Ki67 status, with Ki67-negative ILCs clustering closer to IMC and Ki67-positive ILCs clustering closer to IC (Figure 3A).

**Figure 3:**
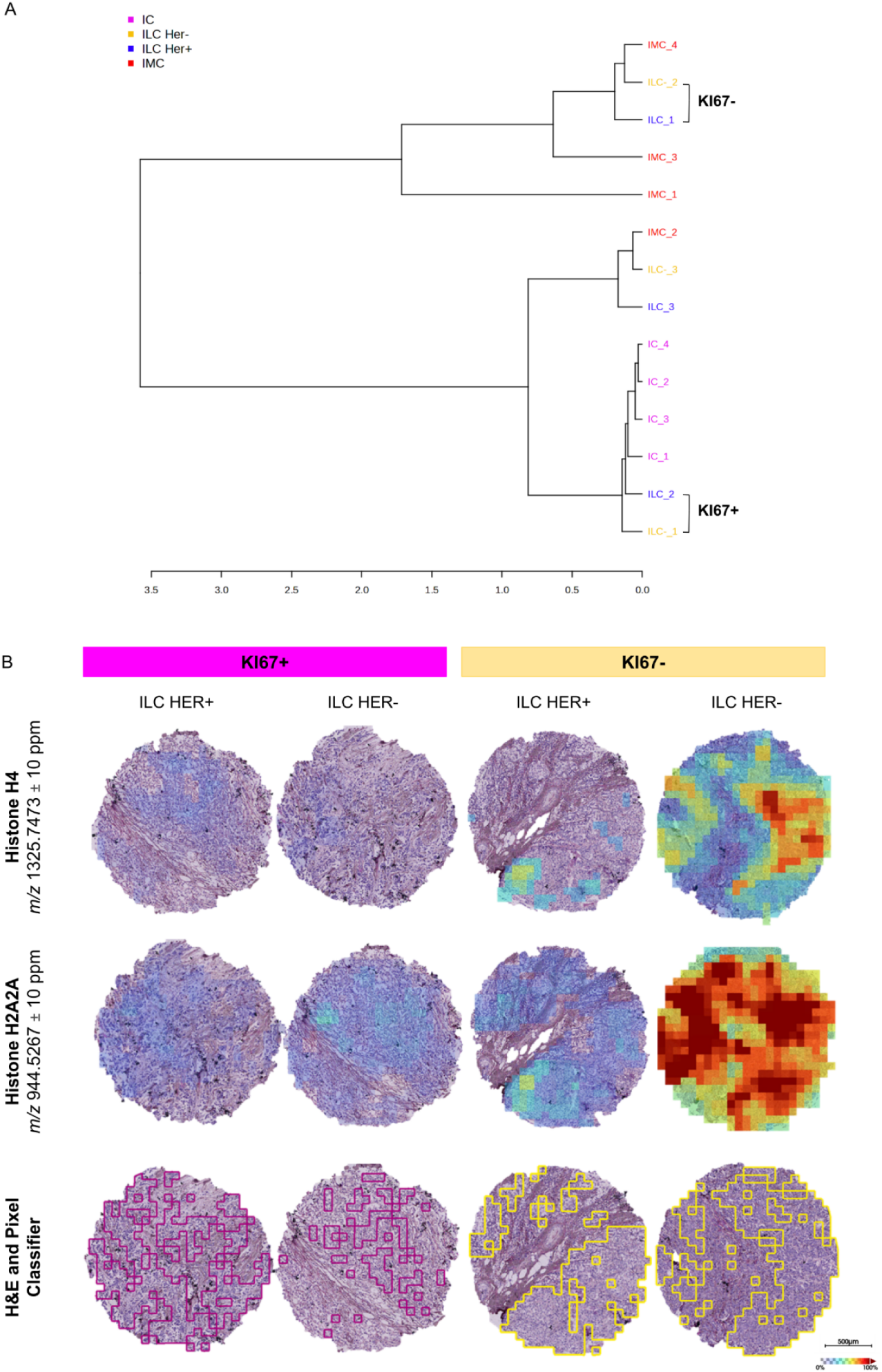
Ki67 drives proteomic differences in ILC: A. Hierarchical clustering of the four tumor conditions (IC, ILC-HER2+, ILC-HER2-, and IMC) with the ILC class stratified for the Ki67 marker. B. MALDI-MSI spatial distribution of histone H4 and histone H2A2A across Ki67-driven ILC tissues further supports the role of Ki67 in discriminating distinct proteomic profiles. H&E and pixel classifier-based annotations for ILC-Ki67+ (in purple) and Ki67- (in yellow) are reported at the bottom. Weak denoising applied.

To further investigate the drivers of this distribution, ROC analysis between ILC conditions stratified by Ki67 identified 26 discriminatory peptides compared to the 31 obtained considering HER2-based stratification. Among them, two histones, histone H4 (*m*/*z* 1325.7473) and histone H2A2A (*m*/*z* 944.5267), were identified using the library created from tissue nESI-LC-MS/MS analysis (Table S5) and matched what Caprioli et al. previously reported. The spatial distribution of these histones is mainly found in areas with high cell density, as seen from the pixel classifier-based annotations, and their expression showed significantly higher intensity under Ki67 conditions (Figure 3B).

This result highlights how, within the ILC subtype, the proliferative state can be a more significant functional driver of proteomic heterogeneity than the receptor status, emphasizing how the tumor’s biological behavior can prevail over classification based solely on receptors.

The increased expression of histones in Ki67-ILCs is consistent with their biological function and aligns with clinical observations in ILC, where Ki67 shows unique biological and prognostic relevance compared to other BC subtypes. Specifically, relatively low Ki67 values (≤4–5%) are sufficient to stratify ILC patients by risk, indicating that even small changes in the proliferative index correspond to markedly different molecular states.^48^ In this context, the combination of low or absent Ki67 expression with high histone levels is more likely to indicate a structured quiescent state, with more compact and epigenetically controlled chromatin, rather than an increase in replicative activity.^49^

Overall, this exploratory analysis does not identify proteins uniquely associated with a single tumor subtype but instead reveals protein expression trends that reflect tumor invasiveness and proliferative state. Consistent with the lipidomic and glycomic layers, the ILC subtype emerges as the most heterogeneous among those analyzed, reinforcing the idea that this tumor entity exhibits intrinsic molecular complexity that manifests at different omics levels.

### Multiomics Integration across Histopathological Subtypes of BC

The integration of the three omics levels analyzed via MALDI-MSI was performed to explore potential molecular signatures among different BC subtypes. As a first step, a correlation analysis was performed to gain a comprehensive overview of the results (Table S7).

Figure 4A reveals the main correlations, both positive (red) and negative (blue), between the molecules at the three levels that appear to define well-defined clusters. The correlation matrix shows a complex block structure, suggesting that the features are not completely independent of one another. Specifically, areas of high positive correlation are noted between lipids and glycans (top right and center) and between lipids/glycans and peptides (top left).

**Figure 4:**
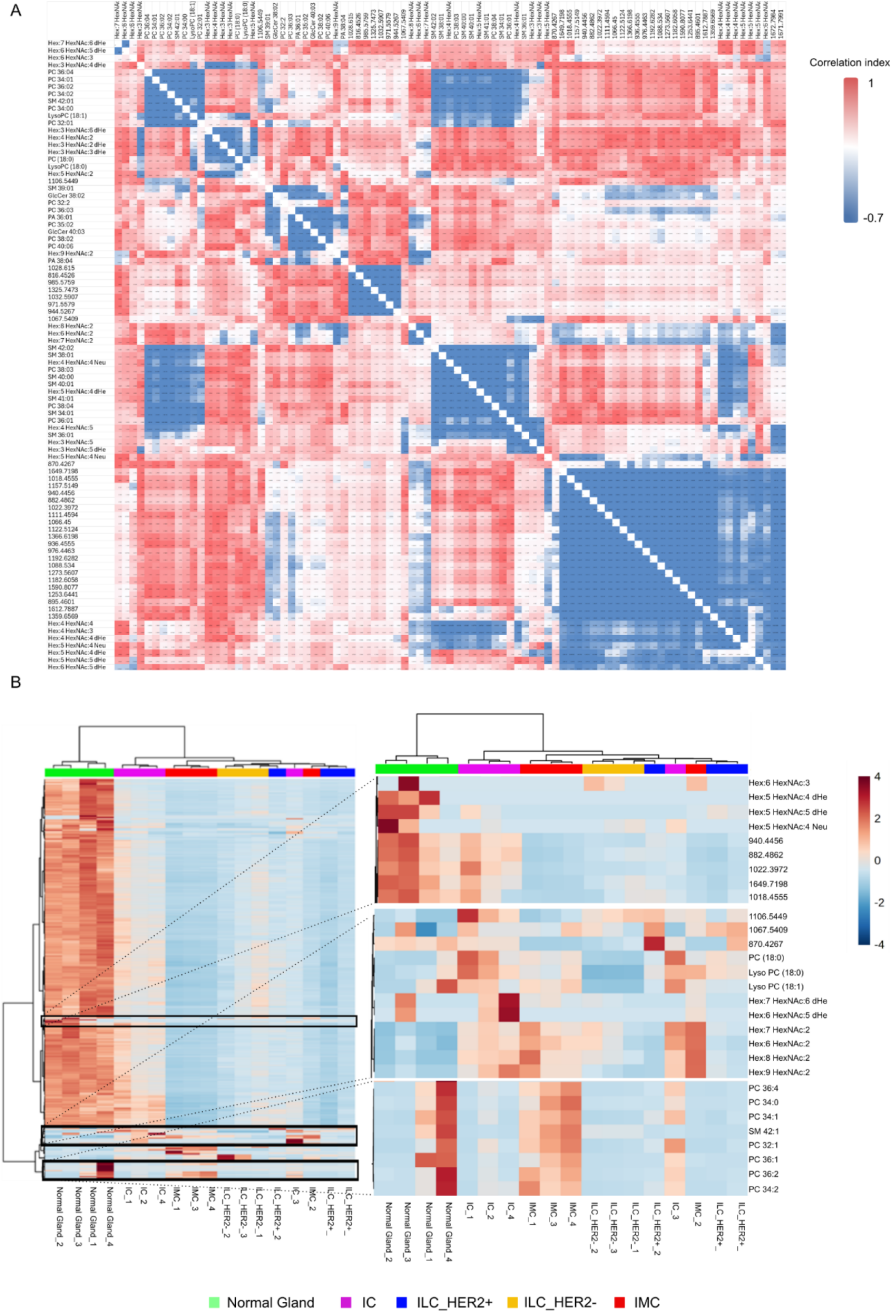
Multiomics integration of lipidomic, N-glycomic, and proteomic layers: A. Correlation matrix of integrated lipidomic, glycomic, and proteomic features from the multiomic analysis. Red indicates positive correlation, while blue indicates negative correlation. B. The hierarchical clustering heatmap generated from integrated lipidomic, glycomic, and proteomic features reveals the molecular stratification of samples across histopathological classes, including BC subtypes and normal gland tissue. The enlarged panel highlights the features contributing most to sample separation across BC classes.

Given this molecular complexity, we expected that it might also reflect the class distinction. Therefore, we performed hierarchical clustering that considered both the grouping between features and different pathological conditions.

The integration of the three omics levels into the heatmap (Figure 4B) supports the presence of distinctive modules with similar or opposing molecular states within the same pathological conditions, as suggested by the correlation analysis. Specifically, Figure 4A reveals a negative correlation between high-mannose glycans and a phospholipid cluster characterized by PC (32:0), PC (34:0), and PC (36:0), compared to a protein cluster located in the bottom right (*m*/*z* 970.4267–1359.6569). The heatmap further supports this relationship by showing a different relative abundance of these molecules under healthy and highly invasive conditions. In contrast, a positive correlation is observed between the LysoPCs, high-mannose glycans, and complex glycans (Hex:7HexNAc:6dHex and Hex:6HexNAc:5dHex), a pattern also observed in the heatmap. Biologically, positive correlations may indicate molecules potentially involved in the same metabolic pathways or coregulated by the same stimulus, rather than proteins involved in lipid and glycan synthesis, modification, or trafficking. Moreover, negative correlation clusters observed between lipids/glycans and peptides may suggest a functional switch or a different cell status.

Beyond molecular associations, the integrated heatmap allows for the separation between the neoplastic conditions and the healthy conditions (Figure 4B). Within the tumor condition, the heatmap shows a structured pattern that enables the discrimination of the four neoplastic subclasses. The presence of two main macrocategories is evident: one is dominated by high molecular expression in healthy samples, and a second, more heterogeneous, is predominantly associated with tumor profiles. The healthy-associated macro class appears to be largely driven by the proteomic layer, with a minor contribution from lipids and glycans, suggesting that the proteomic layer may play a key role in distinguishing between healthy and pathological conditions. The tumor-associated cluster displays greater expression variability and a general upregulation of molecular features.

While the multiomics approach improves healthy-tumor separation compared to single-layer analysis, the distinction between ILC-HER+ and ILC-HER– seems less evident. This effect may reflect the dominant weight of the proteomic layer, which shows differences among ILC classes prevalently driven by Ki67 and may exert the strongest influence on global clustering. Moreover, an attenuated differentiation between ILC tumors according to HER status was observed in glycans,^50,51^ which may be linked to the discoid architecture of ILC cells due to the absence or reduced expression of the glycoprotein E-cadherin (Figure 2). The lack of this protein may limit glycan-mediated cell–cell interactions. This result suggests that HER2-driven modulation within the ILC class may be only minimally reflected in the proteome and glycome layers. In contrast, these architectural distortions might contribute to the heterogeneity and dysregulation observed in ILC classes at the lipidomic layer, leading to significant remodeling of plasma membrane lipids^35^ (Figure 1).

Overall, the multiomics integration suggests feature-sample correlations between the three omics layers, indicating a potentially interconnected lipid, N-glycan, and protein regulatory landscape. Moreover, this approach enables the separation of healthy from neoplastic tissue and supports the discrimination of the major tumor phenotypes, while also highlighting the intrinsic molecular overlap and heterogeneity of ILC subtypes.

This sequential multiomics workflow adopted includes multiple processing steps that may potentially affect analyte localization and signal integrity within the tissue. A comprehensive evaluation of these aspects has been previously reported for this protocol.^28^ In the present work, we focused on applying this established workflow by incorporating a recently developed matrix for lipidomics characterization,^29^ rather than revalidating the protocol itself. Nonetheless, it is important to note that cumulative tissue processing may introduce biases, such as partial analyte delocalization or signal attenuation, which should be considered when interpreting spatial molecular patterns. Future studies on dedicated and larger cohorts could further quantify these effects in the context of newly integrated modalities.

While the current study utilizes a multiomics framework, the integration strategies implemented are comparatively simple. These methods were intentionally selected to ensure robustness and basic biological interpretability, particularly in light of the limited sample size and the exploratory nature of the study. More advanced integrative methodologies, such as latent variable models or multiblock approaches,^52–54^ could provide a more comprehensive characterization of cross-omics relationships. However, they generally require larger cohorts to ensure stability and to avoid overfitting. On that basis, future studies on expanded data sets will enable the application of these advanced frameworks, potentially yielding deeper insights into the molecular mechanisms under investigation.

## Conclusion

In this work, we present the application of a multiomics MALDI-MSI protocol for the spatial analysis of lipids, N-glycans, and peptides on the same slide containing a BC TMA. Due to its exploratory and proof-of-concept design, the findings of this study are considered hypothesis-generating and necessitate further structural and mechanistic validation within larger, independent cohorts. Our preliminary data confirm, both in the individual layers and in their integration, a clear distinction between healthy and tumor tissues, in agreement with previous literature.^38,55–57^

At the lipidomic level, an upregulation of LysoPC and PC was observed in *in situ* tumor lesions compared to invasive ones, while SM displayed the opposite trend. Within ILC subtypes, lipidomic clustering revealed a clear separation between HER2-positive and HER2-negative tumors. A similar distinction was observed in the glycomic layer, suggesting that the HER2 status significantly influences both the lipid and glycomic profiles of ILCs; however, this separation did not emerge in the proteomic analysis. However, these observations are predicated on descriptive spatial associations and must not be construed as evidence of direct mechanistic or causal relationships between the HER2 status and the identified molecular patterns.

Glycomic analysis further highlights an enrichment in high-mannose glycans in IC and IMC, while ILC presented a more attenuated and heterogeneous glycan profile. Proteomic analysis revealed a global protein downregulation in tumor conditions compared to healthy tissue, with ILC stratification primarily driven by Ki67 expression rather than HER2 status, reinforcing the biological heterogeneity associated with this subtype.

Finally, multiomics integration confirmed protein downregulation as a general feature of tumor tissues and identified lipids and glycans as the main contributors to tumor subtype discrimination. To note, the limited sample size and commercial TMA-based design may restrict the generalizability of these findings and may not comprehensively represent the intratumor heterogeneity that is actually present in patient-derived cohorts. Further research is necessary to validate these results across larger, independent cohorts and diverse clinical patient series. Additionally, exploring the potential therapeutic implications of these findings could pave the way for more effective treatment strategies customized to specific tumor subtypes.

## Supplementary Material





## Notes

Descriptive analysis of molecular differences between breast cancer subtypes and normal glandular tissue; lipidomic characterization of breast cancer subtypes; spatial distribution of the N-glycan Hex:5 HexNAc:4 in ILC tissues stratified by HER2 status; matrix deposition parameters used for each omic layer; PNGase F and Trypsin deposition parameters used for N-glycomic and proteomic digestion, respectively; putative annotations of lipid species, N-glycans, and tryptic peptides; data set of cores used for the current analysis (PDF)
Correlation matrix of integrated lipidomic, glycomic, and proteomic features from the multiomics analysis (XLSX)

## Abbreviation

ACN: acetonitrile
ANOVA: analysis of variance
ATT: 6-Aza-2-Thiothymine
AUC: area under curve
BC: breast cancer
Cer: ceramides
CHCA: α-Cyano-4-hydroxycinnamic acid
DAHC: diammonium hydrogen citrate
DIA-PASEF: Data Independent Acquisition-Parallel Accumulation-Serial Fragmentation
DTT: DL-dithiothreitol
ER: estrogen receptor
EtOH: ethanol
FA: formic acid
FFPE: formalin-fixed paraffin-embedded
GlcCer: glycosylated ceramides
GUA: guanidinium chloride
H&E: hematoxylin and eosin
H_2_O: water
HER2: human epidermal growth factor 2
HPLC: high-performance liquid chromatography
IAA: iodoacetamide
IC: intraductal carcinoma
ILC: invasive lobular carcinoma
IMC: invasive medullary carcinoma
LC-MS: liquid chromatography–mass spectrometry
LysoPC: lysophosphatidylcholines
MALDI-MSI: Matrix Assisted Laser Desorption/Ionization Mass Spectrometry Imaging
MeOH: methanol (MeOH)
MS: mass spectrometry
NH_4_HCO_3_: ammonium bicarbonate
nLC–ESI–MS/MS: nano electrospray ionization liquid chromatography tandem mass spectrometry
PC: phosphatidylcholines
PCA: principal component analysis
ppm: part per million
PR: phosphorus red
PR: progesterone receptor
RMS: root-mean-square
ROC: receiver operating characteristic
SM: sphingomyelins
TFA: trifluoroacetic acid
TIF: tumor interstitial fluids
TMA: tissue microarray
TME: tumor microenvironment
TNM: tumor, node, metastasis;
