# Supplementary material for: Sequential MALDI-MSI-Based Multiomics Reveals Spatial Lipid, Glycan, and Tryptic Peptide Signatures in Breast Tumor Histopathology
Source: Anal Chem. 2026 May 6;98(19):14503–15. doi: 10.1021/acs.analchem.6c01243 (PMC13191728; doi:10.1021/acs.analchem.6c01243)
Supplement: Supplementary file 1 [file ac6c01243_si_001.pdf]

Sequential MALDI-MSI-Based Multi-Omics Reveals Spatial Lipid, Glycan, and Tryptic Peptide Signatures in Breast Tumour Histopathology

Seyed M. J. Seyed Golestan[1]†, Nicole Monza[2]†, Farnaz Fatahian[1], Lisa Pagani[2], Mohammad A. AS`habi[1], Hossein Behboudi[1], Andrew Smith[2], Alireza Ghassempour[1]*, and Vanna Denti[2].*

[1]Medicinal Plants and Drug Research Institute, Shahid Beheshti University, Tehran, Iran, 1983969411

[2]University of Milano Bicocca, Department of Medicine and Surgery, Proteomics and Metabolomics Unit, Vedano al Lambro, Italy, 20854

Table of Contents

Figure S1: Descriptive analysis of molecular differences between breast cancer subtypes and normal glandular tissue.

Figure S2: Lipidomic characterisation of breast cancer subtypes.

Figure S3: Spatial distribution of the N-glycan Hex:5 HexNAc:4 in ILC tissues stratified by HER2 status.

Table S1: Matrix deposition parameters used for each omic layer.

Table S2: PNGase F and Trypsin deposition parameters used for n-glycomic and proteomic digestion.

Table S3: Putative annotations of lipid species.

Table S4: Putative annotations of N-glycans.

Table S5: Putative annotations of tryptic peptides.

Table S6: Dataset of cores used for the current analysis.

Table S7: Entire correlation matrix of integrated lipidomic, glycomic and proteomic features from the multi-omics analysis. Blue indicates negative correlation, red indicates positive correlation. (XLSX).

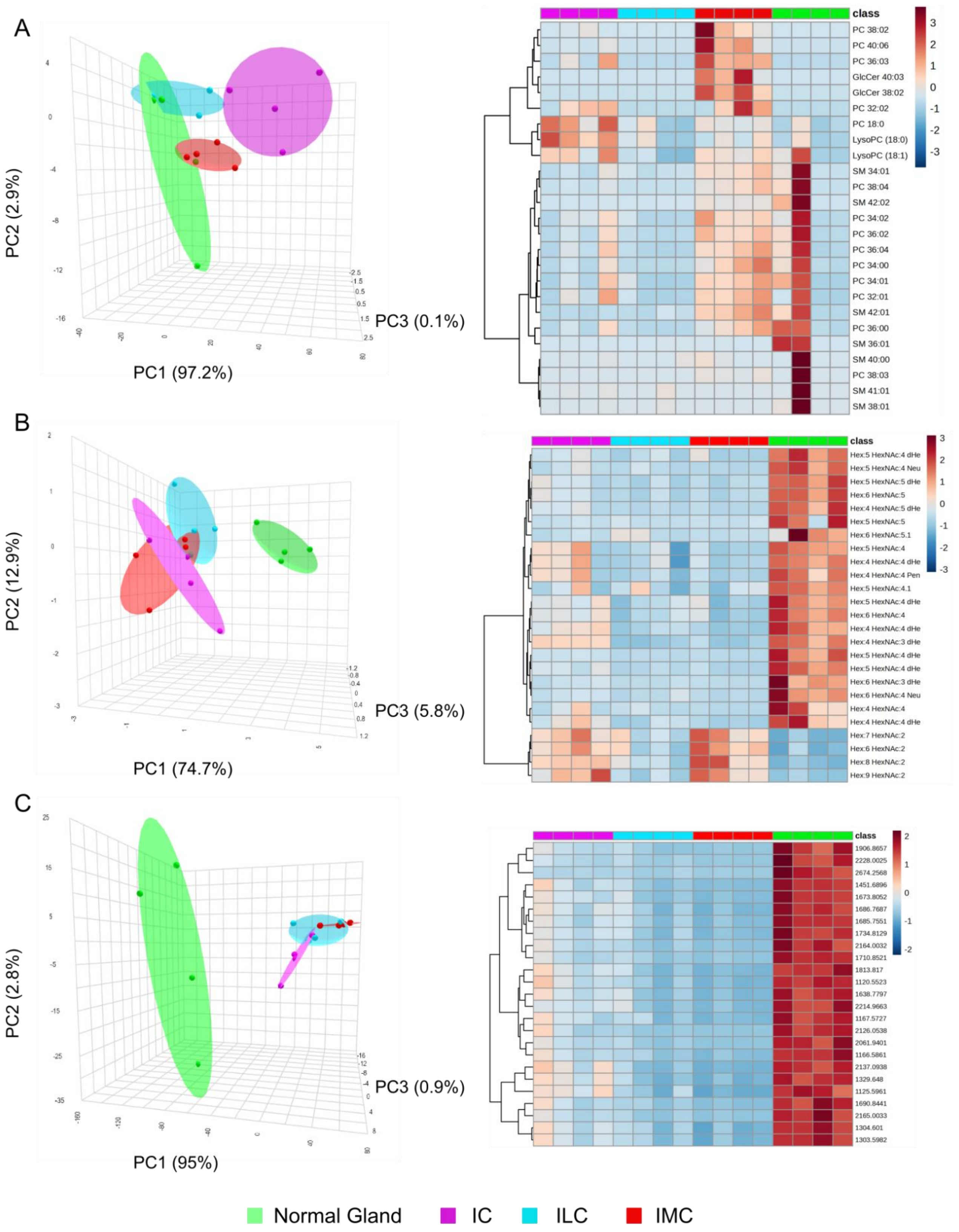

Supplementary Figure S1: Descriptive analysis of molecular differences between breast cancer subtypes and normal glandular tissue.

(A) Lipidomics, (B) N-glycomics, and (C) proteomics PCA and hierarchical clustering highlight a clear separation between normal gland and tumour tissues, with additional subtype-specific molecular stratification across the three omic layers.

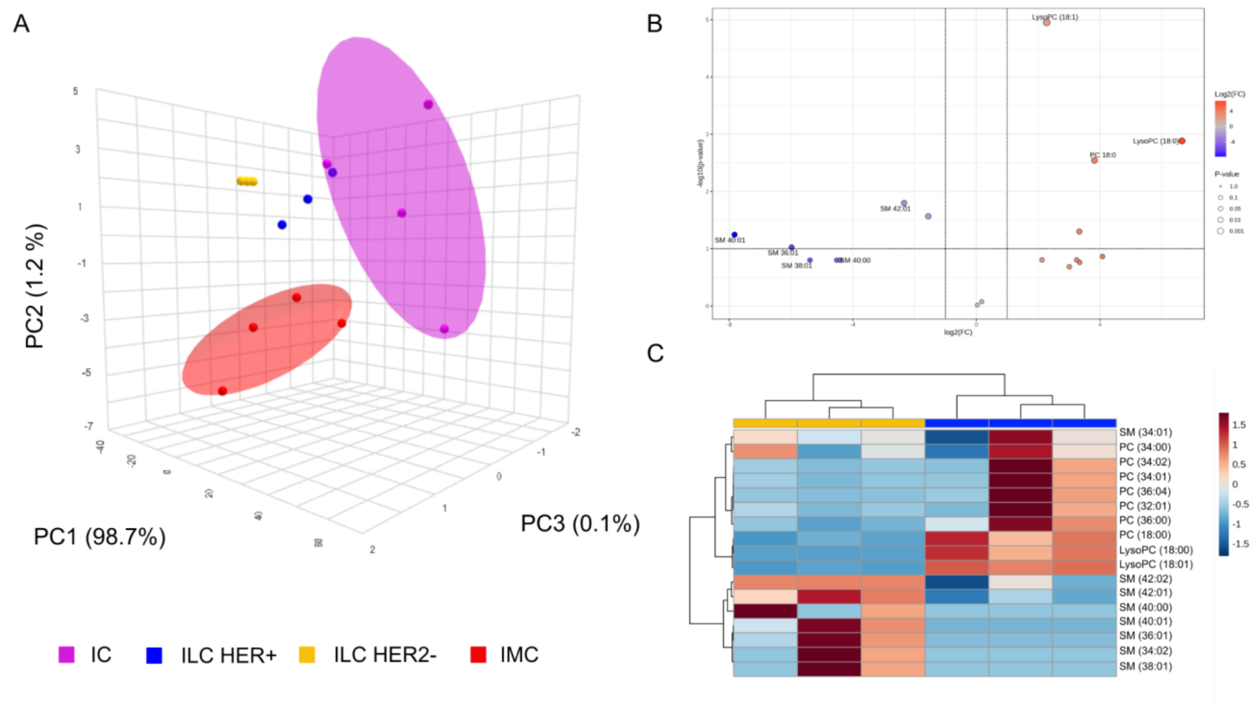

Supplementary Figure S2: Lipidomic characterisation of breast cancer subtypes.

(A) PCA of lipidomic profiles highlights a clear separation among BC subtypes, with additional stratification of ILC according to HER2 status. (B) Volcano plot comparing ILC HER2+ and HER2− indicates a limited but specific set of differentially expressed lipids. (C) Heatmap focused on ILC samples confirms HER2-associated lipid modulation.

A

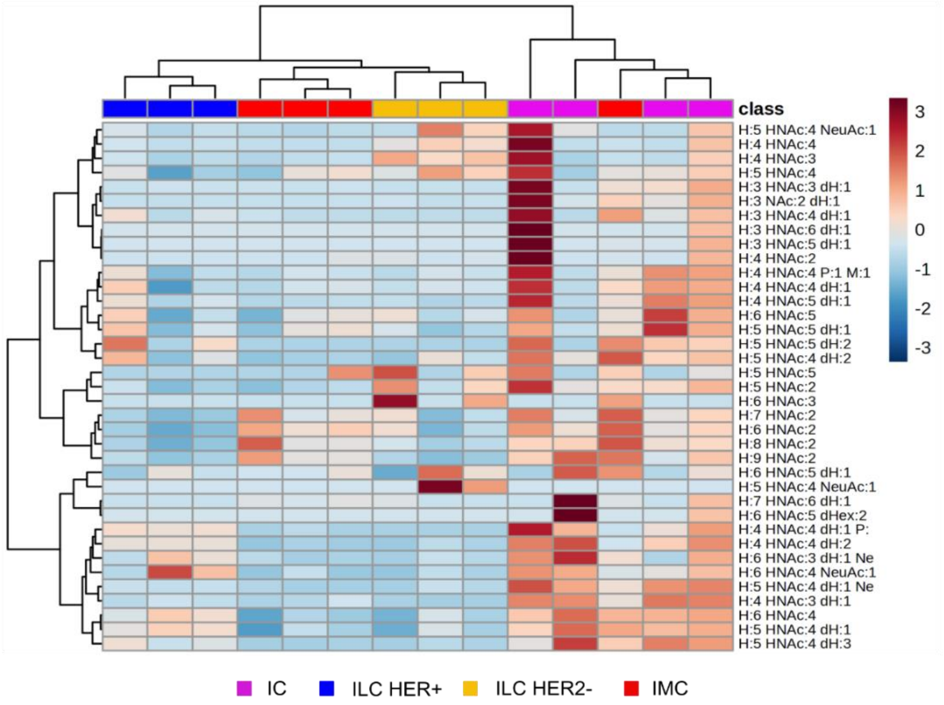

B

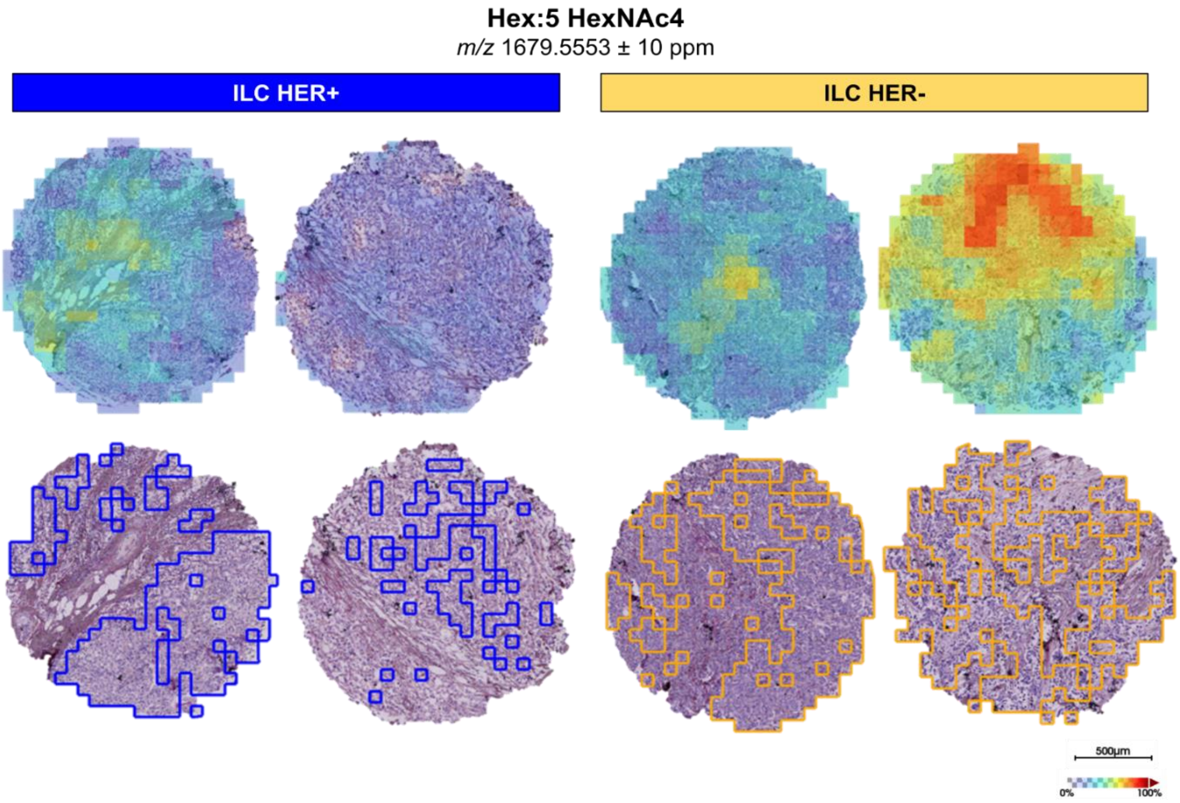

Supplementary Figure S3: Spatial distribution of the N-glycan Hex:5 HexNAc:4 in ILC tissues stratified by HER2 status.

Hierarchical clustering heatmap of the top ANOVA-selected N-glycans reveals in situ and invasive-specific glycan signatures. (B) MALDI-MSI spatial visualisation (top) of the N-glycan Hex:5 HexNAc:4 (*m/z* 1679.5553, AUC: 0.8) shows a higher expression in ILC HER2+ compared to ILC HER2− tissues. H&E staining and PC-based segmentation (bottom) highlight the cell-rich regions in ILC HER2+ (blue) and ILC HER2− (orange). Colour scale intensity: transparent (less intense) to red (more intense). Size scale: 500 µm.

Parameter	*Lipids*	*N-Glycan*	*Tryptic-Peptides*
nozzle temperature (°C)	85	75	75
number of passes	6	4	4
flow rate (mL/min)	0.15	0.12	0.12
velocity (mm/min)	1100	1200	1200
Track spacing (mm)	2	3	3
Pressure (psi)	10	10	10

Table S1: Matrix deposition parameters used for each omic layer.

Parameter	*PNGase F*	*Trypsin*
heat bed temperature (°C)	37	37
height of the needle (mm)	45	45
distance between spray lines (mm)	1.5	2
speed of movement (mm/s)	150	160
number of spray cycles	15	15
matrix density (μL/cm²)	1.2	1.2

Table S2: PNGase F and Trypsin deposition parameters used for n-glycomic and proteomic digestion respectively.

m/z signal	*Lipid identification*	*Formula*	*Ion Notation*	*Error (ppm)*
522.3529	LysoPC 18:1	C26H52NO7P	[M+H]+	-4.732
524.3686	LysoPC 18:0	C26H54NO7P	[M+H]+	-4.726
538.3479	PC 18:0	C26H52NO8P	[M+H]+	-4.576
544.3362	LysoPC 18:1	C26H52NO7P	[M+Na]+	-2.114
546.3508	LysoPC 18:0	C26H54NO7P	[M+Na]+	-3.982
703.5718	SM 34:1	C39H79N2O6P	[M+H]+	-4.386
725.5537	SM 34:1	C39H79N2O6P	[M+Na]+	-4.305
730.5355	PC 32:2	C40H76NO8P	[M+H]+	-3.63
731.603	SM 36:1	C41H83N2O6P	[M+H]+	-4.263
732.5507	PC 32:1	C40H78NO8P	[M+H]+	-4.257
754.5326	PC 32:1	C40H78NO8P	[M+Na]+	-4.133
758.5655	PC 34:2	C42H80NO8P	[M+H]+	-5.199
759.6342	SM 38:1	C43H87N2O6P	[M+H]+	-4.284
760.5817	PC 34:1	C42H82NO8P	[M+H]+	-4.497
762.5953	PC 34:0	C42H84NO8P	[M+H]+	-7.137
780.5477	PC 34:2	C42H80NO8P	[M+Na]+	-4.739
782.5641	PC 36:4	C44H80NO8P	[M+H]+	-6.85
784.5793	PC 34:0	C42H84NO8P	[M+Na]+	-4.353
786.5967	PC 36:2	C44H84NO8P	[M+H]+	-5.143
787.6653	SM 40:1	C45H91N2O6P	[M+H]+	-4.413
788.6123	PC 36:1	C44H86NO8P	[M+H]+	-5.154
800.5785	PC 36:3	C44H82NO9P	[M+H]+	-1.894
804.6064	PC 36:1	C44H86NO9P	[M+H]+	-6.038
806.5593	PC 36:3	C44H82NO8P	[M+Na]+	-9.551
810.5957	PC 38:4	C46H84NO8P	[M+H]+	-6.166
811.6659	SM 40:0	C45H93N2O6P	[M+Na]+	-0.58
812.6105	PC 38:3	C46H86NO8P	[M+H]+	-7.261
813.6812	SM 42:2	C47H93N2O6P	[M+H]+	-3.91
814.6273	PC 38:2	C46H88NO8P	[M+H]+	-5.81
815.6956	SM 42:1	C47H95N2O6P	[M+H]+	-5.454
818.587	GlcCer 40:3	C46H85NO8	[M+K]+	-4.473
835.6636	SM 42:2	C47H93N2O6P	[M+Na]+	-3.304

Table S3: Putative annotations of lipid species obtained with Metaboscape. *m/z* signal, lipid identification, lipid formula and mass error in ppm are reported.

m/z signal	*N-glycan identification*	*Formula*	*Ion Notation*	*Error ppm*
1095.3488	Hex:3 HexNAc:2 dHex:1	C40H68N2O30	[M+K]+	0.156
1111.3438	Hex:4 HexNAc:2	C40H68N2O31	[M+K]+	0.306
1273.3966	Hex:5 HexNAc:2	C46H78N2O36	[M+K]+	0.325
1298.4282	Hex:3 HexNAc:3 dHex:1	C48H81N3O35	[M+K]+	0.980
1314.4231	Hex:4 HexNAc:3	C48H81N3O36	[M+K]+	0.018
1435.4494	Hex:6 HexNAc:2	C52H88N2O41	[M+K]+	0.017
1460.481	Hex:4 HexNAc:3 dHex:1	C54H91N3O40	[M+K]+	-0.655
1501.5076	Hex:3 HexNAc:4 dHex:1	C56H94N4O40	[M+K]+	0.158
1517.5025	Hex:4 HexNAc:4	C56H94N4O41	[M+K]+	0.056
1558.5291	Hex:3 HexNAc:5	C58H97N5O41	[M+K]+	0.729
1597.5022	Hex:7 HexNAc:2	C58H98N2O46	[M+K]+	-0.093
1638.5288	Hex:6 HexNAc:3	C60H101N3O46	[M+K]+	0.009
1663.5604	Hex:4 HexNAc:4 dHex:1	C62H104N4O45	[M+K]+	1.209
1663.5814	Hex:5 HexNAc:4	C62H104N4O46	[M+Na]+	-8.565
1664.5682	Hex:4 HexNAc:4 Pent:1 Me:1	C62H105N4O45	[M+K]+	-1.007
1679.5553	Hex:5 HexNAc:4	C62H104N4O46	[M+K]+	-0.261
1704.587	Hex:3 HexNAc:5 dHex:1	C64H107N5O45	[M+K]+	0.308
1720.5819	Hex:4 HexNAc:5	C64H107N5O46	[M+K]+	0.130
1759.5551	Hex:8 HexNAc:2	C64H108N2O51	[M+K]+	0.285
1808.5979	Hex:4 HexNAc:4 NeuAc:1	C67H111N5O49	[M+K]+	3.515
1809.6183	Hex:4 HexNAc:4 dHex:2	C68H114N4O49	[M+K]+	6.439
1810.6261	Hex:4 HexNAc:4 dHex:1 Pent:1 Me:1	C68H115N4O49	[M+K]+	4.846
1825.6132	Hex:5 HexNAc:4 dHex:1	C68H114N4O50	[M+K]+	0.336
1825.6342	Hex:6 HexNAc:4	C68H114N4O51	[M+Na]+	-9.213
1866.6398	Hex:4 HexNAc:5 dHex:1	C70H117N5O50	[M+K]+	-0.040
1882.6347	Hex:5 HexNAc:5	C70H117N5O51	[M+K]+	0.679
1907.6663	Hex:3 HexNAc:6 dHex:1	C72H120N6O50	[M+K]+	-1.433
1921.6079	Hex:9 HexNAc:2	C70H118N2O56	[M+K]+	-0.422
1970.6507	Hex:5 HexNAc:4 NeuAc:1	C73H121N5O54	[M+K]+	-0.092
1971.6711	Hex:5 HexNAc:4 dHex:2	C74H124N4O54	[M+K]+	-5.396
1992.6327	Hex:5 HexNAc:4 NeuAc:1	C73H120N5O54	[M+K]+	-1.303
2028.6926	Hex:5 HexNAc:5 dHex:1	C74H123N7O54	[M+Na]+	7.083
2028.7136	Hex:6 HexNAc:5	C76H127N5O56	[M+Na]+	-8.772
2044.6875	Hex:6 HexNAc:5	C76H127N5O56	[M+K]+	-0.304
2116.7087	Hex:5 HexNAc:4 dHex:1 NeuAc:1	C79H131N5O58	[M+K]+	-0.368
2117.7291	Hex:5 HexNAc:4 dHex:3	C80H134N4O58	[M+K]+	-5.872
2138.6906	Hex:5 HexNAc:4 dHex:1 NeuAc:1	C79H130N5O58	[M+K]+	-0.328
2154.6855	Hex:6 HexNAc:4 NeuAc:1	C79H130N5O59	[M+K]+	-6.894
2155.6389	Hex:6 HexNAc:3 dHex:1 NeuAc:1 Su:1	C77H128N4O61S	[M+K]+	5.495
2174.7505	Hex:5 HexNAc:5 dHex:2	C82H137N5O59	[M+K]+	4.112
2190.7454	Hex:6 HexNAc:5 dHex:1	C82H137N5O60	[M+K]+	-1.007
2261.7462	Hex:5 HexNAc:4 NeuAc:2	C84H138N6O62	[M+K]+	-0.018
2262.7666	Hex:5 HexNAc:4 dHex:2 NeuAc:1	C85H141N5O62	[M+K]+	-5.252
2336.8033	Hex:6 HexNAc:5 dHex:2	C88H147N5O64	[M+K]+	-1.440
2555.8776	Hex:7 HexNAc:6 dHex:1	C96H160N6O70	[M+K]+	-1.409

Table S4: Putative annotations of N-glycans obtained with Metaspace software. *m/z* signal, n-glycans identification, and ion notations are reported.

m/z signal	*m/z nLC-ESI-MSMS*	*Protein accession*	*Error ppm*
785.384	785.3883	CO1A2_HUMAN	-5.475
809.4325	809.4329	CO1A2_HUMAN	-0.49417
816.4526	816.4534	H2B1D_HUMAN	-0.97985
868.4229	868.4247	CO1A1_HUMAN	-2.07272
868.4586	868.4612	CO1A2_HUMAN	-2.9938
870.4267	870.4233	MDHC_HUMAN	3.906145
880.4959	880.4969	MACF1_HUMAN	-1.13572
882.4862	882.4781	IF4E_HUMAN	9.178698
886.433	886.4384	CO1A1_HUMAN	-6.09179
895.4601	895.4607	CO1A2_HUMAN	-0.67005
898.4691	898.4733	CD53_HUMAN	-4.6746
908.4897	908.4922	PGFRB_HUMAN	-2.75181
936.4555	936.4628	ODPA_HUMAN	-7.79529
940.4456	940.4455	ALBU_HUMAN	0.106333
944.5267	944.5283	H2A2A_HUMAN	-1.69397
950.4656	950.4615	VTDB_HUMAN	4.313694
965.4992	965.5025	EZRI_HUMAN	-3.41791
971.5579	971.5596	ASSY_HUMAN	-1.74976
976.4463	976.4454	POTEE_HUMAN	0.921711
982.4346	982.4375	K1C18_HUMAN	-2.95184
985.5759	985.5758	ITIH2_HUMAN	0.101464
1018.456	1018.452	UBA1_HUMAN	3.731154
1022.397	1022.404	RL37L_HUMAN	-6.65099
1028.615	1028.618	KAPCG_HUMAN	-2.7221
1032.591	1032.592	H31T_HUMAN	-1.25897
1045.559	1045.558	DESP_HUMAN	1.434641
1053.541	1053.538	FXL20_HUMAN	3.037385
1062.542	1062.541	FA12_HUMAN	1.035254
1064.55	1064.55	NDUS1_HUMAN	-0.09394
1066.45	1066.442	PLEC_HUMAN	7.59535
1067.541	1067.549	HVC33_HUMAN	-7.96216
1069.572	1069.57	AT1A1_HUMAN	1.495928
1078.537	1078.532	FAS_HUMAN	4.543212
1079.563	1079.565	MOES_HUMAN	-1.66734
1081.582	1081.589	TPD54_HUMAN	-6.47196
1084.561	1084.565	PDIA3_HUMAN	-3.78032
1088.534	1088.532	VIME_HUMAN	1.837337
1090.56	1090.559	PLCA_HUMAN	1.008657
1095.562	1095.562	CO1A2_HUMAN	-0.09128
1096.511	1096.513	GRB2_HUMAN	-2.27995
1098.478	1098.469	PLEC_HUMAN	8.193219
1105.571	1105.564	TENA_HUMAN	5.87935
1106.545	1106.549	PRS6A_HUMAN	-3.34373
1107.546	1107.546	6PGD_HUMAN	0.180579

1109.557	1109.559	SDHA_HUMAN	-1.53214
1111.592	1111.586	PYC_HUMAN	5.397692
1113.519	1113.528	ACTN3_HUMAN	-7.7232
1115.539	1115.538	RS27A_HUMAN	1.075715
1116.572	1116.578	RL18_HUMAN	-4.65709
1120.552	1120.561	EZRI_HUMAN	-7.40701
1121.565	1121.561	ITAL_HUMAN	3.65562
1122.512	1122.507	RS21_HUMAN	4.81066
1125.596	1125.598	PGBM_HUMAN	-1.33263
1131.534	1131.535	RS19_HUMAN	-0.79538
1133.513	1133.509	TCPH_HUMAN	3.2642
1138.563	1138.569	COCA1_HUMAN	-5.00628
1139.565	1139.567	DOCK6_HUMAN	-2.28157
1145.542	1145.543	SPF45_HUMAN	-1.04754
1154.559	1154.567	COCA1_HUMAN	-7.18884
1155.563	1155.567	SPTN1_HUMAN	-3.11535
1157.515	1157.51	PEDF_HUMAN	3.887654
1160.545	1160.549	BAIP2_HUMAN	-2.92965
1163.587	1163.586	DDX3Y_HUMAN	0.859412
1164.591	1164.589	PLEC_HUMAN	2.060813
1166.586	1166.59	SKT_HUMAN	-3.68596
1167.573	1167.57	FHR2_HUMAN	2.398143
1168.494	1168.499	FHL1_HUMAN	-4.10783
1170.552	1170.553	FBP1L_HUMAN	-0.59801
1182.606	1182.609	LMNA_HUMAN	-2.875
1183.551	1183.554	DYHC1_HUMAN	-2.36576
1184.49	1184.492	RL4_HUMAN	-1.51964
1192.628	1192.627	CRYAB_HUMAN	0.838485
1196.575	1196.576	TAP2_HUMAN	-1.33715
1197.565	1197.562	PSD13_HUMAN	2.421587
1198.573	1198.573	RHOQ_HUMAN	-0.08343
1200.544	1200.547	G3BP1_HUMAN	-2.49886
1201.556	1201.558	ITIH2_HUMAN	-1.91418
1207.57	1207.567	PGBM_HUMAN	2.81558
1214.603	1214.602	STIP1_HUMAN	0.823315
1223.611	1223.612	CO1A2_HUMAN	-0.24518
1225.565	1225.575	LUM_HUMAN	-8.89378
1226.569	1226.567	EIF3C_HUMAN	1.875153
1235.609	1235.609	TRA2B_HUMAN	-0.08093
1245.593	1245.603	CFAH_HUMAN	-7.86768
1249.661	1249.661	SYMC_HUMAN	0.160043
1251.605	1251.604	ITB4_HUMAN	1.038667
1253.644	1253.642	PA2G4_HUMAN	1.356049
1259.594	1259.583	SAHH_HUMAN	8.177308
1261.59	1261.592	PDIA5_HUMAN	-1.98162
1266.595	1266.593	AL3A2_HUMAN	1.026375
1267.671	1267.671	AHNK_HUMAN	-0.15777

1273.561	1273.566	ECM1_HUMAN	-4.24006
1274.592	1274.582	FA12_HUMAN	8.15954
1275.65	1275.65	FLNB_HUMAN	-0.07839
1276.63	1276.629	TRFE_HUMAN	0.548319
1278.552	1278.544	HDGR2_HUMAN	6.569975
1280.616	1280.626	AHNK_HUMAN	-7.34016
1285.605	1285.603	PERE_HUMAN	1.244552
1288.608	1288.616	ATS4_HUMAN	-5.58739
1289.652	1289.65	LRP1_HUMAN	1.938511
1290.599	1290.589	PRS8_HUMAN	7.825883
1297.609	1297.605	BAP31_HUMAN	3.082603
1298.61	1298.614	HNRPL_HUMAN	-3.15721
1300.628	1300.638	COCA1_HUMAN	-7.84231
1303.598	1303.597	HSP7C_HUMAN	0.690397
1304.601	1304.598	TYB4_HUMAN	2.529515
1309.59	1309.603	PERI_HUMAN	-9.6976
1316.6	1316.598	EIF3I_HUMAN	2.126694
1320.661	1320.66	MMP14_HUMAN	0.757197
1322.555	1322.546	ZYX_HUMAN	6.805058
1323.619	1323.622	CSPG2_HUMAN	-2.56871
1325.747	1325.75	H4_HUMAN	-1.8103
1326.582	1326.588	TM87A_HUMAN	-4.59826
1328.643	1328.636	TMOD3_HUMAN	5.042766
1329.648	1329.65	VAT1_HUMAN	-1.20332
1337.675	1337.676	SODE_HUMAN	-0.14951
1340.592	1340.59	CALD1_HUMAN	1.566475
1342.642	1342.629	ALBU_HUMAN	9.086647
1350.626	1350.633	ANXA6_HUMAN	-4.8866
1351.633	1351.626	CAPG_HUMAN	5.178947
1359.657	1359.656	PDIA3_HUMAN	0.809028
1366.62	1366.621	ACTB_HUMAN	-0.95125
1368.626	1368.622	ACTN4_HUMAN	3.287979
1368.626	1368.63	HNRLL_HUMAN	-2.92263
1375.627	1375.617	IGHA1_HUMAN	6.978686
1384.621	1384.625	KHDR1_HUMAN	-2.81665
1385.633	1385.625	RAB8B_HUMAN	6.134416
1407.628	1407.641	TENA_HUMAN	-9.59051
1408.668	1408.675	TAGL_HUMAN	-4.89822
1412.673	1412.682	K2C8_HUMAN	-6.22928
1413.665	1413.674	AGAL_HUMAN	-6.36639
1415.662	1415.663	RNPL1_HUMAN	-0.56511
1422.669	1422.676	E41L3_HUMAN	-5.20146
1423.607	1423.607	SIAE_HUMAN	0.070244
1424.626	1424.629	K2C6A_HUMAN	-1.89523
1426.722	1426.725	JUPI2_HUMAN	-1.82236
1429.667	1429.665	DHB4_HUMAN	1.328982
1431.677	1431.68	SYTC_HUMAN	-1.95574

1441.679	1441.669	RS10L_HUMAN	7.144499
1442.68	1442.676	CO4A1_HUMAN	2.911257
1444.66	1444.656	PHB1_HUMAN	3.045708
1447.677	1447.676	LAMA5_HUMAN	0.690762
1448.683	1448.68	DHX16_HUMAN	1.725709
1449.691	1449.692	SPTB2_HUMAN	-0.3449
1451.69	1451.688	ZA2G_HUMAN	1.239936
1455.667	1455.677	DDX17_HUMAN	-6.93835
1459.687	1459.689	CLH1_HUMAN	-1.50717
1471.674	1471.673	ROA3_HUMAN	0.611549
1473.667	1473.664	PML_HUMAN	2.103601
1475.684	1475.691	COCA1_HUMAN	-4.47248
1477.741	1477.745	PIMT_HUMAN	-2.3008
1490.691	1490.686	HRG_HUMAN	3.75666
1491.695	1491.697	PRP8_HUMAN	-1.47483
1493.744	1493.732	K1C10_HUMAN	8.43525
1499.732	1499.722	NUP58_HUMAN	6.667905
1508.706	1508.708	WASC4_HUMAN	-1.92217
1509.707	1509.703	CFAH_HUMAN	2.980719
1510.716	1510.72	EIF3D_HUMAN	-2.64774
1513.72	1513.727	FIBG_HUMAN	-4.3601
1514.728	1514.731	S10AG_HUMAN	-2.1786
1515.721	1515.719	ZYX_HUMAN	1.649382
1526.702	1526.706	EF1D_HUMAN	-2.68552
1530.689	1530.683	CO6A2_HUMAN	4.377132
1531.687	1531.686	TRFE_HUMAN	0.78345
1532.697	1532.7	PDC6I_HUMAN	-1.69635
1533.676	1533.676	RL5_HUMAN	-0.0652
1540.717	1540.722	IGHA1_HUMAN	-3.24523
1546.792	1546.793	PRKRA_HUMAN	-1.22835
1549.667	1549.654	HNRPK_HUMAN	8.905217
1553.714	1553.71	CHD4_HUMAN	2.445759
1561.789	1561.789	NUD16_HUMAN	-0.12806
1562.789	1562.787	CO1A2_HUMAN	0.959823
1563.722	1563.714	COCA1_HUMAN	5.307876
1568.763	1568.765	ACL6A_HUMAN	-1.46612
1570.636	1570.641	LGMN_HUMAN	-3.56542
1585.766	1585.773	RL27A_HUMAN	-4.66649
1590.808	1590.802	TPP1_HUMAN	3.457375
1591.695	1591.696	MYH10_HUMAN	-0.75391
1596.756	1596.756	ARHG1_HUMAN	0.187881
1601.742	1601.75	CH60_HUMAN	-4.80724
1602.745	1602.745	RL18A_HUMAN	-0.43675
1607.748	1607.748	GHDC_HUMAN	-0.0622
1608.742	1608.748	SPTN1_HUMAN	-3.66745
1609.732	1609.739	LAMB2_HUMAN	-4.28641
1612.789	1612.788	CAH1_HUMAN	0.186013

1613.798	1613.8	PSB3_HUMAN	-1.30128
1614.783	1614.78	TRFL_HUMAN	2.229406
1615.778	1615.774	IBP2_HUMAN	2.042365
1619.772	1619.778	U2AF2_HUMAN	-3.519
1623.719	1623.726	VASP_HUMAN	-4.06473
1625.724	1625.726	ACTN3_HUMAN	-0.98418
1631.793	1631.797	TPM2_HUMAN	-2.88026
1636.767	1636.774	DFFA_HUMAN	-4.15451
1637.768	1637.762	FIBA_HUMAN	3.663535
1638.78	1638.778	RALA_HUMAN	0.915316
1641.75	1641.751	MYH9_HUMAN	-0.67002
1648.731	1648.731	NUCL_HUMAN	-0.30326
1649.72	1649.709	POSTN_HUMAN	6.546609
1652.823	1652.819	MTX1_HUMAN	2.722622
1655.794	1655.789	UBA1_HUMAN	2.596949
1664.771	1664.768	IGHG2_HUMAN	1.381574
1665.77	1665.778	GELS_HUMAN	-4.6825
1665.783	1665.781	CPSF6_HUMAN	1.56083
1669.82	1669.813	VTNC_HUMAN	3.71299
1670.814	1670.816	RASK_HUMAN	-1.67583
1671.799	1671.796	PKP3_HUMAN	1.734661
1672.798	1672.796	AASD1_HUMAN	1.614064
1673.805	1673.81	FLNC_HUMAN	-2.92745
1674.807	1674.807	DYLT1_HUMAN	0
1677.769	1677.764	NPL4_HUMAN	2.980158
1677.777	1677.78	P4K2A_HUMAN	-1.43046
1678.781	1678.771	NACAM_HUMAN	5.837605
1685.755	1685.742	PPME1_HUMAN	7.652416
1686.769	1686.785	LRGUK_HUMAN	-9.84121
1690.774	1690.776	CO1A1_HUMAN	-1.41947
1695.746	1695.742	NONO_HUMAN	2.653706
1697.752	1697.751	PLP2_HUMAN	0.883522
1698.809	1698.81	KPCA_HUMAN	-0.58865
1699.754	1699.76	RA1L3_HUMAN	-3.47108
1700.747	1700.755	RL10_HUMAN	-4.40981
1701.756	1701.772	ENDD1_HUMAN	-9.10815
1702.76	1702.762	TXD17_HUMAN	-1.05711
1710.852	1710.851	NIPS1_HUMAN	0.876757
1712.771	1712.777	FBN1_HUMAN	-3.03601
1713.788	1713.787	ANXA3_HUMAN	0.466802
1716.812	1716.805	MP2K3_HUMAN	4.077341
1718.815	1718.813	RAB2A_HUMAN	0.756336
1720.869	1720.865	ANM1_HUMAN	2.150081
1723.734	1723.744	F120A_HUMAN	-6.09139
1729.755	1729.754	ACTN1_HUMAN	1.098422
1734.813	1734.807	VIME_HUMAN	3.170381
1735.829	1735.832	FOXA1_HUMAN	-1.90111

1742.734	1742.73	FIBB_HUMAN	2.123106
1751.826	1751.836	GDIR1_HUMAN	-5.47997
1756.773	1756.76	IF2B_HUMAN	7.456911
1758.829	1758.816	ACTN4_HUMAN	7.391338
1758.84	1758.839	MTL26_HUMAN	0.341134
1766.75	1766.757	PGBM_HUMAN	-4.24507
1767.814	1767.831	LIMS2_HUMAN	-9.6163
1769.813	1769.81	RS21_HUMAN	1.921111
1773.799	1773.798	SYSM_HUMAN	0.563762
1775.873	1775.873	APOB_HUMAN	0.05631
1776.87	1776.873	NSF1C_HUMAN	-1.96975
1782.817	1782.821	RS4X_HUMAN	-2.29973
1797.849	1797.844	IDHC_HUMAN	2.55862
1798.85	1798.848	PALLD_HUMAN	1.223005
1799.847	1799.851	GFPT1_HUMAN	-1.94461
1811.862	1811.858	ANXA2_HUMAN	2.318063
1812.816	1812.818	1433G_HUMAN	-0.93777
1813.869	1813.868	PURA_HUMAN	0.551308
1816.852	1816.849	MDHC_HUMAN	1.651211
1817.858	1817.851	SEPT9_HUMAN	3.68567
1819.861	1819.859	AHNK_HUMAN	0.76929
1820.863	1820.872	PDLI4_HUMAN	-5.38204
1821.862	1821.848	MYH10_HUMAN	7.464947
1828.825	1828.83	ESYT1_HUMAN	-2.78867
1832.854	1832.86	SODE_HUMAN	-3.05534
1833.903	1833.906	BIP_HUMAN	-1.5268
1836.864	1836.873	ADH1G_HUMAN	-4.62743
1841.847	1841.859	CRKL_HUMAN	-6.9495
1844.84	1844.841	ALS_HUMAN	-0.59626
1847.867	1847.866	APOB_HUMAN	0.541165
1848.854	1848.856	IF4A2_HUMAN	-1.35219
1849.857	1849.869	PDIA5_HUMAN	-6.27072
1851.858	1851.865	ROA2_HUMAN	-3.88797
1855.885	1855.899	AMPB_HUMAN	-7.48963
1856.893	1856.903	GRP75_HUMAN	-5.43916
1870.848	1870.847	CRK_HUMAN	0.801776
1888.912	1888.929	MYH9_HUMAN	-8.84099
1906.866	1906.877	MTA2_HUMAN	-5.97836
1910.898	1910.915	VWF_HUMAN	-8.68694
1922.924	1922.933	SPTB2_HUMAN	-4.73235
1923.892	1923.904	SYLC_HUMAN	-6.02941
1940.876	1940.865	PML_HUMAN	5.513005
1946.867	1946.887	ANXA4_HUMAN	-9.81054
1961.974	1961.975	EF2_HUMAN	-0.6626
1965.921	1965.914	IBP4_HUMAN	3.713286
1983.954	1983.968	SPTB2_HUMAN	-6.90535
1992.924	1992.913	ACTN1_HUMAN	5.419202

1999.923	1999.932	SRRT_HUMAN	-4.30015
2003.969	2003.98	PLAK2_HUMAN	-5.33938
2038.961	2038.962	TOIP1_HUMAN	-0.4414
2056.086	2056.088	CO3_HUMAN	-1.2159
2056.966	2056.957	TENA_HUMAN	4.132317
2061.94	2061.944	COPE_HUMAN	-2.08541
2072.97	2072.977	RNPS1_HUMAN	-3.18383
2073.963	2073.968	DJC10_HUMAN	-2.41084
2080.961	2080.948	KCC2B_HUMAN	6.535484
2081.957	2081.964	HUWE1_HUMAN	-3.45827
2088.996	2089.003	CAZA1_HUMAN	-3.49449
2094.92	2094.91	ABCD3_HUMAN	4.439331
2095.921	2095.911	NPTN_HUMAN	4.866618
2104.074	2104.077	TCPA_HUMAN	-1.2357
2105.997	2106.011	H1BP3_HUMAN	-6.64764
2115.114	2115.127	COCA1_HUMAN	-6.47715
2116.108	2116.115	SYFB_HUMAN	-3.44972
2126.054	2126.057	VIME_HUMAN	-1.5992
2137.094	2137.109	PLEK_HUMAN	-7.06562
2164.003	2164.022	IDHC_HUMAN	-8.59511
2214.966	2214.972	RCN2_HUMAN	-2.66369
2220.955	2220.969	SPTB2_HUMAN	-6.61873
2228.003	2228.018	ALDOA_HUMAN	-6.91197
2519.207	2519.186	CALR_HUMAN	8.455113
2674.257	2674.276	GABT_HUMAN	-7.17951
2727.312	2727.289	PDXD1_HUMAN	8.139951
2891.389	2891.41	ITA2_HUMAN	-7.12455

Table S5: Putative annotations of proteins obtained with the in-house generated library using nLC-ESI-MS/MS. *m/z* signal, *m/z* nLC-ESI-MS/MS, protein accession, and error ppm are reported.

Type	*ER*	*PR*	*HER2*	*Ki67*	*TNM*	*Stage*
Normal	+	+	0	–	–	–
Normal	+	+	0	–	–	–
Normal	+	+	0	–	–	–
Normal	+	+	0	–	–	–
IC	5%	–	3+	5%	TisN0M0	0
IC	5%	–	3+	5%	TisN0M0	0
IC	60%	60%	3+	10%	TisN0M0	0
IC	–	–	3+	10%	TisN0M0	0
ILC (HER2+)	100%	3%	3+	–	T2N0M0	–
ILC (HER2+)	100%	50%	3+	5%	T4N1M0	–
ILC (HER2−)	80%	50%	0	–	T4N1M0	–
ILC (HER2−)	40%	40%	0	20%	T4N0M0	–
IMC	60%	–	0	15%	T3N0M0	IIB
IMC	–	–	0	–	T3N0M0	IIB

IMC	–	–	0	20%	T4N0M0	IIIB
IMC	–	–	0	20%	T4N0M0	IIIB

Table S6: Dataset of cores used for the current analysis. Type of lesion (normal gland, Intraductal carcinoma (IC), Invasive Lobular Carcinoma (ILC) positive or negative for HER2, Invasive Medullary Carcinoma (IMC)), estrogen receptor (ER) status, progesterone receptor (PR) status, Human Epidermal growth factor Receptor 2 (HER2) status, Ki67 status, tumour nodule metastasis (TNM) grade and stage are reported.
